# Five Years after the Fort McMurray Wildfire: Prevalence and Correlates of Low Resilience

**DOI:** 10.3390/bs12040096

**Published:** 2022-03-30

**Authors:** Medard Kofi Adu, Ejemai Eboreime, Reham Shalaby, Adegboyega Sapara, Belinda Agyapong, Gloria Obuobi-Donkor, Wanying Mao, Ernest Owusu, Folajinmi Oluwasina, Hannah Pazderka, Vincent I. O. Agyapong

**Affiliations:** 1 Department of Psychiatry, University of Alberta, Edmonton, AB T6G 2B7, Canada; medard@ualberta.ca (M.K.A.); eboreime@ualberta.ca (E.E.); rshalaby@ualberta.ca (R.S.); asapara@ualberta.ca (A.S.); bagyapon@ualberta.ca (B.A.); obuobido@ualberta.ca (G.O.-D.); wmao2@ualberta.ca (W.M.); eowusu2@ualberta.ca (E.O.); folajinm@ualberta.ca (F.O.); hannah@ualberta.ca (H.P.); 2 Global Psychological E-Health Foundation, Edmonton, AB T6G 2B7, Canada; 3 Department of Psychiatry, Dalhousie University, Halifax, NS B3H 2E2, Canada

**Keywords:** disaster, wildfires, predictors, mental health, PTSD, resilience

## Abstract

Background: The Fort McMurray wildfire of 3 May 2016 was one of the most devastating natural disasters in Canadian history. Although resilience plays a crucial role in the daily functioning of individuals by acting as a protective shield that lessens the impact of disasters on their mental well-being, to date little is known about the long-term impact of wildfires on resilience and associated predictors of low resilience. Objectives: The objective of the study was to assess the prevalence and predictors of resilience among residents of Fort McMurray five years after the wildfires. Method: This was a quantitative cross-sectional study. A self-administered online survey which included standardized rating scales for resilience (BRS), anxiety (GAD-7), depression (PHQ-9), and post-traumatic stress disorder (PTSD)(PCL-C) was used to determine the prevalence of resilience as well as its demographic, clinical, and wildfire-related predictors. The data were collected between 24 April and 2 June 2021 and analyzed using the Statistical Package for Social Sciences (SPSS) version 25 using univariate analysis with a chi-squared test and binary logistic regression analysis. Results: A total of 186 residents completed the survey out of 249 who accessed the online survey, producing a response rate of 74.7%. Most of the respondents were females (85.5%, 159), above 40 years of age (81.6%, 80), employed (94.1%, 175), and in a relationship (71%, 132). Two variables—having had PTSD symptoms (OR = 2.85; 95% CI: 1.06–7.63), and age—were significant predictors of low resilience in our study. The prevalence of low resilience in our sample was 37.4%. Conclusions: Our results suggest that age and the presence of PTSD symptoms were the independent significant risk factors associated with low resilience five years after the Fort McMurray wildfire disaster. Further research is needed to enhance understanding of the pathways to resilience post-disaster to identify the robust predictors and provide appropriate interventions to the most vulnerable individuals and communities.

## 1. Introduction

Fort McMurray is located within the Regional Municipality of Wood Buffalo. The city is made up of several neighborhoods which include: Abasand Heights, Beacon Hill, Gregoire, the Lower Townsite, Parsons Creek, Thickwood Heights, Timberlea, and Waterways (RMWB 2015a). The city is sited in the Northeastern part of the province of Alberta. Classified as an urban service area, the city according to data from Alberta’s Municipal Service branch has a population of about 82,724 [1] (Government of Alberta 2015). 

The Fort McMurray wildfire of 3 May 2016 was one of the most devastating natural disasters that ever happened in Canadian history. This disaster, also referred to as the Wood Buffalo Wildfire or Horse River Wildfire was dubbed ‘‘The Beast’’ in the popular media [1] due to its unpredictable nature. It began on 1 May 2016 and burned through almost 600,000 hectares of land [2], with the destruction of about 2400 homes, it led to a record largest and most rapid Canadian wildfire evacuation of 88,000 residents on 3 May 2016 until it was declared under control on 5 July 2016. It was the most expensive natural disaster in Canadian history; the Insurance Bureau of Canada 2016 indicated that the cost of the Fort McMurray wildfire was estimated at USD 3.6 billion in insured losses [3]. The aftermath of the evacuation of the Fort McMurray wildfire saw many individuals displaced as a result of damage to their homes or otherwise made unsuitable for habitation by the fire [4]. It also led to unemployment and job losses due to damage caused to businesses and the closure of the few local ones. Aside from the physical damage to properties, the community was faced with social, psychological, and emotional challenges after the wildfire [4,5].

Wildfires stem from a combination of multiple factors and key among them are dry fuels, ignition, and weather [2]. All these factors are greatly influenced by climatic changes in the form of a rise in temperature, drought, and heat warnings with the resultant effect of drier fuels that lead to greater intensity of wildfires [2]. Uniquely, the consequence of wildfires persists over an extended period and hence causes great disruption of daily functioning and eventually leads to an imbalanced psychological state and overall well-being [6,7]. With the intensity of wildfire disasters increasing progressively over the past 200 years, it is estimated that approximately two-thirds of victims have feared for their lives, with a resultant increase in the prevalence of post-traumatic stress disorder (PTSD) afterward [1]. Though natural disasters affect a great number of people, the impact of the experience differs from person to person [8]. Before the return to pre-disaster levels of functioning, the impact felt may range from short term to long term and may involve a series of reactions, such as problems with day-to-day functioning and clinical syndromes such as PTSD [9].

Epidemiological studies have found that a majority of people (60% to 80%) will be impacted with at least one major traumatic event in their lifetime, and yet comparably, the lifetime prevalence of PTSD remains low, at approximately 8% [10,11]. From a broader perspective, studies reported an increase in the rate of suicides months and years after a natural disaster [12,13]. This is of particular concern because an individual’s ability or inability to deal with the physical and psychological adversities during and after a devastating disaster is an important factor in the determination of long-term mental health outcomes of the affected persons [4].

Resilience has been described as a person’s capacity to handle inimical life experiences based on their mindset, resourcefulness, and social support from family, friends, and the community at large. Greater resilience, in the form of dependence on social support systems such as family and friends, has been associated with mitigating the negative consequences of wildfires [14,15]. Notwithstanding the mental health challenges encountered from experiences of disasters, resilience plays a crucial role in the daily functioning of individuals by acting as a protective shield that lessens the impact of the disaster on their mental well-being [16,17]. On the other hand, a greater risk of psychopathology post-disaster is associated with non-resilience that manifests in the forms of low social support, low socioeconomic status, and poor interpersonal relationships [13,18]. Researchers have described resilience as being made up of different protective factors that act together [19].

The five protective factors that make up the core of resilience include meaningfulness, perseverance, self-reliance, existential aloneness, and equanimity [20]. Meaningfulness is when an individual acknowledges that their life has purpose and meaning, while perseverance is the capacity to continue even amid setbacks. Individuals who are self-reliant acknowledge their strengths and rely on those strengths to direct their actions in times of difficulty. Existential aloneness is described as the ability to recognize that while some experiences can be shared, a person must be able to face and manage other experiences on their own; in possessing equanimity, the individual is said to have a stable view of life, mental calmness, and evenness of temper, especially in difficult situations [8,21].

Although there is no established standardized definition of resilience, it is defined based on two key concepts: positive adaptation and adversity [22,23]. Theoretically, there are divergent views on whether resilience is conceptualized as a personality trait or as a process [23,24]. Resilience is seen as a trait mostly characterized by features such as hardiness, positive self-esteem, optimism, and quality problem-solving skills [25,26]. As a process, and with the tendency to change over a period, it is usually a dynamic process that enables positive adaptation relative to significant adversity [27]. Individuals can effectively manage adversity based on these characteristics of resilience. There is enough evidence in the literature to support the fact that resilience may help improve the overall psychological well-being of an individual and enable a better recovery from inimical situations like natural disasters [28]. This is supported by studies of college students exposed to multiple traumatic situations which revealed that high trait resilience was negatively associated with the symptoms of PTSD [29]. Individuals with higher resilience mostly make use of their personality characteristics to minimize the hazardous effect on their health outcomes of stress placed on them by disasters [30]. Research suggests that predictors of the resilience of an individual are often determined by a wide range of factors which stems from genetics, biological, psychological, and environmental factors [31,32,33]. Identifying the predictors of resilience for individuals and communities affected by natural disasters of a higher magnitude, such as the Fort McMurray wildfires, is an essential priority for psychiatric research in that the understanding of positive adaptation to stressors may help with prevention plans and also positively influence the required interventions aimed at helping the individuals at risk to recover from these inimical situations and their related psychological problems [34]. Previous studies have sought to examine the long-term effects of the Fort McMurray wildfires of 2016 by evaluating the predictors and prevalence of likely mental conditions such as major depressive disorder (MDD), generalized anxiety disorder (GAD), and PTSD [1,4]. The primary aim of this study is to contribute further to the understanding of the psychological impact of the wildfires by looking at the predictors and correlates of resilience five years after the Fort McMurray wildfires.

## 2. Methods

### 2.1. Study Design, Sample Size, and Institutional Review Board Approval

This study employed a cross-sectional survey design which was used to collect quantitative data through an online-based self-administered questionnaire. Participants were included if they were aged 18 years and above, residents of Fort McMurray at the time of the wildfire and its evacuation processes, and they received the online-based self-administered questionnaire. Temporary visitors, as well as those living in Fort McMurray for less than a year, were excluded. A total sample of 249 surveys was received and the exclusion of incomplete responses yielded 186 complete surveys. Online informed consent was obtained from all study participants after they had reviewed the online information leaflets concerning the study, following the Declaration of Helsinki [35]. This study was carried out as per the recommendations and approval of the University of Alberta Review and Ethics Board (Pro00066054).

### 2.2. Sample Size Estimation

With a population of 111,687 as of the 2018 census, a 95% confidence interval, and a ±5% margin of error, the sample size needed for prevalence rate estimates for PTSD was 383.

### 2.3. Data Collection and Analysis

Demographic and clinical information, as well as wildfire exposure and support-related information, was collected with a data collection form designed for this study. The study utilized socio-demographic and wildfire-related variables such as age, employment, and area of residence of respondents before the wildfires; we also considered the areas with greater volumes of damaged properties, where residents were on the day of the evacuation, and where respondents lived after the fire. Moreover, variables such as whether respondents witnessed homes being destroyed by the wildfire and whether they were fearful for their lives and/or those of their family and friends during the evacuation were also considered. Clinical variables included the history of mental health diagnoses such as anxiety, depression, and PTSD. Finally, we included variables such as the quality of support received during the evacuation from family and friends, insurance companies, the Red Cross, and the Government of Alberta, and whether respondents had received mental health counseling or were willing to receive mental health counseling after the wildfire. The scales used in this study were duly validated and had been previously used to collect data from a large number of residents, six months and 18 months after the Fort McMurray wildfires. Further details are also provided in related publications [1,36].

The patient health questionnaire (PHQ-9) [37] was used to assess the presence or absence of likely MDD in respondents. The PHQ-9 scoring was performed using the standard recommendation with the threshold for likely depression being met if 5 of the 9 items were checked at least “more than half the days” and either item A or B was checked at least “more than half the days”. A score ≥ 10 denoted moderate-to-severe depression. The reliability and validity of the tool have indicated it as having sound psychometric properties. The internal consistency of the PHQ-9 has been shown to be high. A study involving two different patient populations produced Cronbach alphas of 0.86 and 0.89. As standard self-report measures were used, there was no potential for bias in the data that were collected. Probable anxiety in the respondents was evaluated using the Generalized Anxiety Disorder-7 (GAD-7) scale. The scale is made up of seven self-reported items which are rated on a 4-point Likert-type scale. The scores range from 0 to 2,1 with higher scores indicating severe GAD symptoms [38]. Respondents who had a GAD-7 score of 10 or more were considered to present likely anxiety. In a heterogeneous clinical population, the GAD-7 has superb internal consistency and a one-factor structure [38], implying that its items are all representative of one construct. The GAD-7 has been reviewed as a valid tool for screening and assessing the severity of GAD in clinical research [39]. Regarding the validity and reliability in the Canadian community, the internal consistency and test–retest reliability of the GAD-7 were good (Cronbach α = 0.92; intraclass correlation = 0.83), and it also provided good criterion, construct, factorial, and procedural validity [40]. The survey measured PTSD symptoms using the PTSD Checklist Civilian (PCL-C) [41,42], a self-report scale that measures PTSD presence and severity. The 17-item checklist corresponds to the PTSD symptoms as stated by the Diagnostic and Statistical Manual (DSM-IV). The level of distress produced by each symptom is rated from 1 (not at all) to 5 (extremely). A score ≥ 44 is deemed clinically significant (maximum score = 85). The PCL-C has been shown to have good reliability and convergent validity [41]. The Brief Resilience Scale (BRS) [43] was used to assess resilience as the main outcome variable of the study. BRS is made up of six questions. Items 1, 3, and 5 are positively worded, and Items 2, 4, and 6 are negatively worded. The BRS is scored by reverse coding Items 2, 4, and 6 and finding the mean of the six items. The following instructions are used to administer the scale: “Please indicate the extent to which you agree with each of the following statements by using the following scale: 1 = strongly disagree, 2 = disagree, 3 = neutral, 4 = agree, 5 = strongly agree” [39]. Regarding reliability and validity, Smith et al. (2008) [44] in their results indicate that the BRS has good internal consistency, with Cronbach alphas ranging from 0.80 to 0.90. In addition, test–retest reliability coefficients using ICCs for a two-week interval were fair, ranging from 0.61 to 0.69. The items were tested on undergraduate students, demonstrating its applicability in the university context. For analysis, we compiled normal and high resilience into one category to compare with low resilience.

The scales used have been validated and used in similar research previously conducted in Canada [1].

The data were analyzed using the Statistical Package for Social Sciences (SPSS) version 25 (IBM Corp 2011) [45]. Descriptive statistics for demographic characteristics were reported in numbers and percentages. Cross-tabular univariate analyses with chi-square or Fisher’s exact tests were used to explore the relationship between the categorical variables in the study and whether respondents had high-to-normal or low resilience. Variables with a statistically significant relationship (*p* ≤ 0.05, two-tailed) and variables that trended toward significance (0.05 < *p* < 0.10, two-tailed) with the likelihood of low resilience on univariate analysis were the only variables analyzed using logistic regression modeling. Preliminary checks were conducted to confirm all the assumptions of the logistic regression, including the presence of an adequate sample size and the absence of outliers. Correlation diagnostics were also performed to ensure the absence of multicollinearity, and high inter-correlations among the predictor variables were avoided. Spearman’s correlation coefficients were less than or equal to 0.7 for all included variables, which suggested that no multicollinearity existed among the independent variables. Odds ratios from the binary logistic regression analysis were examined to determine the association between each of the variables in the model and the likelihood of individuals reporting low resilience, controlling for the other variables in the model. For each of the variables, the first variable option was used as the reference against which other variable options were compared.

## 3. Results

Descriptive characteristics of the sample (including sociodemographic, living situation, exposure to disaster, and clinical variables) against respondents’ age groups are presented in Table 1. Their associations with high-to-normal and low resilience and the descriptor variables are shown in Table 2. The results of the logistic regression examining significant predictors of low resilience are shown in Table 3.

Table 1 shows that most of the respondents were females (85.5%, 159), above 40 years of age (81.6%, 80), employed (94.1%, 175); in a relationship (71%, 132), residents of Fort McMurray at the time of the wildfires (89.8%, 167), and living in their own homes (78.0%, 145). Although many reported no significant loss of property or business due to the wildfire (44.1%, 82), most participants reported a high degree of exposure to the wildfire. For example, most were in town during the evacuation (89.8%, 159) and witnessed houses burning (83.6%, 148). Most participants reported daily exposure to television images (80.2%, 142) and frequently reading newspaper and internet articles (85.9%, 152) about the inimical effects of the wildfires. A total of 159 respondents (89.3%) reported being fearful for their lives or their family members’ lives.

For clinical characterization, 48.4% (90) of the respondents reported the absence of a mental health diagnosis history before the wildfires, while 38.7% of the respondents sought mental health counseling in the past year (38.7%), with 52.7% (98) of participants willing to receive mental health counseling. Most participants reported receiving absolute support from a variety of sources, including family and friends (66.3%, 116), the Red Cross (42.9%, 75), the government (32.8%, 57), and insurance companies (48.3%, 84) after the evacuation order was declared. The prevalence of low resilience in our sample was 37.4% (64).

### 3.1. Univariate Analysis

The association between all socio-demographic and wildfire-related variables and the likelihood that respondents had high-to-normal or low resilience is illustrated in Table 2. For clinical variables, we found some merits to include the clinical conditions of MDD, GAD, and PTSD variables based on the measurement scales (PHQ-9, GAD-7, and PCL-C) rather than the history of the condition in the association analysis. The results suggest statistically significant (*p* ≤ 0.05) associations between 12 socio-demographic and clinical variables (i.e., age, current employment status, presence of depression symptoms, presence of anxiety symptoms, likely PTSD, absence of pre-existing mental disorder, current antidepressant therapy, absence of psychiatric medication therapy, received mental health counseling in the past, fearful for your life or those of family/friends during the evacuation, and received sufficient support from family/friend after evacuation). For example, those aged above 40 years, those currently employed, and those who responded yes to having moderate to high depression, moderate to high anxiety, and likely PTSD had higher rates of low resilience compared to respondents with other characteristics within the same variables. Other variables such as having support from family and friends, being fearful for your life or those of family or friends, responding yes to having received mental health counseling and wanting to receive some, responding yes to being on antidepressants, and responding yes to not being on any medication were significantly associated with the resilience variable.

### 3.2. Logistic Regression

The selection of variables to be included as potential predictors in the logistic regression analysis was informed by the results of the chi-square analysis. Specifically, twelve of the variables identified through the chi-square analysis (see Table 2) with significant *p*-values (*p* ≤ 0.05) were entered into a logistic regression model along with two additional variables (sufficient support from your insurers after evacuation and suffered no loss of property) which were near significance (0.05 < *p* < 0.10).

Two variables that were highly correlated with other included variables were thus excluded from the model (i.e., never having received a mental health diagnosis and being on antidepressants).

The full model containing all twelve predictors was significant, x^2^ (df = 17, *n* = 154.79) = 60.78, *p* < 0.001, suggesting that the model was able to distinguish between respondents who reported low resilience and others. The model explained between 31.3% (Cox and Snell R2) and 42.5% (Nagelkerke R2) of the variance. In addition, 79.6% of all cases were correctly classified. However, only 2 of the 12 predictors made unique contributions to the model (age and having PTSD on the PCL-C scale). As indicated in Table 3 and controlling for all characteristics, age was a significant predictor of low resilience. Respondents who were >40 years and who were between 25 and 40 years were less likely to show low resilience compared to those <25 years (OR = 0.025; 95% CI: 0.001–0.559 and (OR = 0.044; 95% CI: 0.002–0.952, respectively). Conversely, respondents who had PTSD symptoms as indicated on the PCL-C scale were about three times more likely to show low resilience compared to respondents who did not show symptoms of PTSD (OR = 2.85; 95% CI: 1.06–7.63), after controlling for other model variables.

Employment, being on medication, having received mental health counseling in the past year, being fearful for your life and those of family and friends during the evacuation, not having lost property, having received support from insurers, and having received support from family and friends were not significantly associated with low resilience when all the factors in the model were controlled for.

## 4. Discussion

The purpose of this study was to enhance the understanding of the psychological impact of the wildfires by evaluating the predictors of resilience and its correlation with PTSD five years after the wildfire.

This study provides new information about the association between resilience and demographic and clinical characteristics while adding to the growing body of evidence on the beneficial effect of resilience [25,46,47]. The multiple regression analysis demonstrated that two variables (age and having PTSD as recorded on the PCL-C scale) showed significant association with low resilience.

In this study, we found that age was positively correlated with resilience and was a statistically significant predictor of low resilience. This suggests that individuals who were <25 years were more likely to present with low resilience compared to those >40 years. This result is consistent with results of a previous study conducted in the New York City area after the 11 September 2001 terrorist attack [48]. In that study, the researchers looked at predictive factors of psychological resilience after the disaster. Older people were reported to be more than three times more likely (OR 3.11) to be resilient compared with individuals < 25 years of age. In other previous studies, it was identified that as people age, they become more resilient [34,49]. Conversely, the results of a study by Yu et al. [50] are inconsistent with our results, as their results indicated that younger students had higher resilience than older students. Other studies also indicated a negative relationship between resilience and age [50,51]. In another study [52], there was no statistical difference between the total resilience score of older patients (142 ± 25) and that of younger patients (138 ± 12, *p* = 0.13) among cancer patients receiving chemotherapy. While it is worth considering the possibility that the potential distress of trauma may manifest in other domains of adjustment [46,53], further research should be conducted to examine the association between age and resilience among victims post disaster [54].

Our results also suggest that having PTSD was correlated with resilience and was a statistically significant predictor of low resilience. This result is consistent with the results of a previous study conducted on firefighters who had high levels of resilience (upper 25th percentile, ≥75); these firefighters seemed to overcome distress and to be protected from the risk of developing PTSD symptoms, but those who displayed lower levels of individual resilience (up to the 75th percentile, <75) were more prone to developing PTSD [55]. This finding explains the importance of individual resilience as a protective factor against PTSD symptoms in firefighters with greater levels of perceived stress [55]. Similarly, in another study, resilience was found to be a significant predictor of PTSD symptom severity and to mediate the effect of childhood trauma on post-traumatic adjustment [56]. Furthermore, our findings are consistent with those of a previous study in which resilience as a trait was found to be associated with PTSD, which implies that individuals with high resilience are at lower risk of developing PTSD [29]. Interestingly, in another study that evaluated psychological resilience in New York City in the aftermath of the September 11 terror attack, although the exposure categories that led to the highest estimates of likely PTSD indicated lower levels of resilience compared to other groups, the relationship between PTSD prevalence and resilience prevalence was far from perfect [57]. For instance, PTSD was about twice as common in individuals who were in the World Trade Center at the time of the attack compared with those who witnessed the attacks in person from outside the World Trade Center [29,57]. We hope that future research will help to bring more meaning into the relationship between PTSD and resilience. 

Surprisingly, our data suggest that having anxiety disorder did not positively correlate with resilience and that it was not a statistically significant predictor of low resilience. This finding is inconsistent with that of a previous report, which found a strong relationship between neuroticism and resilience in healthy young adults [58]. Furthermore, in a previous study, individuals with anxiety-related symptoms or impairments were found to have lower levels of resilience [59]. Our result is also inconsistent with that obtained by other researchers whose data suggested that individuals with a high level of stress are less resilient [60]. The possible explanation may be that, although stressful life events and adversity can have a substantial impact on an individual and can result in the development of psychological problems, some individuals do not develop such illnesses after experiencing stressful life events and are said to be resilient. Thus, resilience as successful adaptation depends on effective responses to environmental challenges and ultimate resistance to the deleterious effects of stress.

In addition, according to the results, having a depressive disorder did not correlate with resilience and it was not a statistically significant predictor of low resilience. The finding is inconsistent with that of a previous study [30], in which the results indicated that resilience was associated with depression among adolescents exposed to the Wenchuan earthquake. That is, individuals with many depressive symptoms showed low levels of resilience, whereas those with higher resilience reported fewer or no symptoms of depression. A possible explanation for their finding is that higher resilience is usually associated with more positive emotions [61,62] and elevated emotional flexibility [63], which increase the capacity for coping successfully with adversity [25,64].

Our study provides a direction for future research that evaluates resilience in the face of adversity. Other similar studies can compare responses to help identify whether there are variations in responses according to geographical region to similar adversities of life. Moreover, the presence of PTSD symptoms may need to be acknowledged and addressed carefully after disasters, as the consequences of these symptoms were significantly linked to lower resilience, which could affect the overall functioning and the ability to mitigate the negative consequences of these disasters, including wildfires. Furthermore, the results of this study suggest that, post-disaster, it is important not only to screen residents for mental-health-related conditions but also to assess their resilience levels and provide help for vulnerable individuals and communities in order to enhance their protective factors for coping with inimical events such as wildfires. Our findings should be interpreted with caution given that the participants less than 25 years of age were fewer in number by far than the rest. Given the channels through which we recruited participants (intermediaries such as government, community agencies, etc.), our sample may not be very reflective of the general Fort McMurray population. It may rather reflect the random distribution of Fort McMurray residents accessible via email through these intermediaries during the pandemic.

### Limitation of the Study

Studies involving post-disaster conditions often present some unavoidable limitations. This study has several limitations that require consideration when interpreting the findings. First, this study was based on a cross-sectional design, which limits the development of the causal relationship between resilience and other variables. Aside from the relatively small sample size for our study, the measurement scales (PCL-C, GAD-7, and PHQ-9) used for the assessment of the respective clinical variables were self-reported by respondents; hence, no formal diagnosis was possible. Our analysis is therefore based only on likely GAD, MDD, and PTSD diagnosis. Moreover, our sample may not be a reflection of the general population of Fort McMurray; it may instead be representative of the distribution of residents who were accessible during the pandemic using our recruitment channels (emails by intermediaries). However, this was the only feasible and ethics-approved means of contact during the peak of the pandemic. Another important limitation involves the correlational nature of the study, post-test-only design, and the absence of baseline data for comparison. This implies that the result of the study is correlational; hence, causal inferences may not be made. The evaluation of resilience in a cross-sectional manner implies that we are assessing an individual’s perception at one point in time. We therefore cannot tell whether these variables would change over time or whether the perception and resilience measured at a point in time would allow for the prediction of a respondent’s behavior in the future. Thirdly, this study only assessed the influence of individual-level factors on resilience; future studies should therefore be conducted to explore the effect of both individual and community-level factors on resilience for disaster-affected regions.

## 5. Conclusions

Our study suggests that the only modifiable risk factor for low resilience five years post-wildfires is the presence of likely PTSD. Thus, widening the scope of treatment interventions for persons with PTSD and other stress-related conditions will potentially enhance the resilience of victims following wildfire disasters. Psychological first aid and population-level psychological interventions such as the use of supportive text messages [65,66,67,68,69,70,71,72] may help mitigate PTSD symptoms and build resilience in individuals impacted by wildfires.

## Figures and Tables

**Table 1:** Demographic profile, clinical characteristics, and support received by the study population.

Variables	≤25 Year *n* (%)	26–40 Year *n* (%)	>40 Year *n* (%)	Total *n* (%)
Gender				
Male	4 (30.8)	5 (6.7)	18 (18.4)	27 (14.5)
Female	9 (69.2)	70 (93.3)	80 (81.6)	159 (85.5)
Employment status				
Employed	8 (61.5)	74 (98.7)	93 (94.9)	175 (94.1)
Unemployed	5 (38.5)	1 (1.3)	5 (5.1)	11 (5.9)
Employment place				
School boards	3 (37.5)	38 (52.1)	46 (49.5)	87 (50.0)
Healthcare industry	2 (25.0)	3 (4.1)	5 (5.4)	10 (5.7)
Keyano College	1 (12.5)	8 (11.0)	11 (11.8)	20 (11.5)
Oil sands industry	1 (12.5)	6 (8.2)	6 (6.5)	13 (7.5)
Municipal or government agency	0 (0.0)	6 (8.2)	7 (7.5)	13 (7.5)
Other	1 (12.5)	12 (16.4)	18 (19.4)	31 (17.8)
Marital status				
Married/partnered/cohabiting	3 (23.1)	59 (78.7)	70 (71.4)	132 (71.0)
Divorced/separated/widowed	0 (0.0)	2 (2.7)	16 (16.3)	18 (9.7)
Single	10 (76.9)	14 (18.7)	12 (12.2)	36 (19.4)
Did respondents reside in Fort McMurray during the 2016 wildfire?				
Yes	9 (69.2)	64 (85.3)	94 (95.9)	167 (89.8)
No	4 (30.8)	11 (14.7)	4 (4.1)	19 (10.2)
Area of residence during the 2016 wildfire				
0–1.0 properties destroyed/km^2^	6 (66.7)	24 (37.5)	46 (48.9)	76 (45.5)
1.1–50.0 properties destroyed/km^2^	1 (11.1)	21 (32.8)	25 (26.6)	47 (28.1)
50.1–300.0 properties destroyed/km^2^	2 (22.2)	19 (29.7)	23 (24.5)	44 (26.3)
Where did respondents live prior to the 2016 Fort McMurray wildfire?				
Own home	9 (69.2)	47 (62.7)	80 (81.6)	136 (73.1)
Renting	4 (30.8)	28 (37.3)	18 (18.4)	50 (26.9)
Where do respondents live now?				
Own home	8 (61.5)	57 (76.0)	80 (81.6)	145 (78.0)
Renting	5 (38.5)	18 (24.0)	18 (18.4)	41 (22.0)
History of mental health diagnosis from a health professional?				
Depression	3 (23.1)	24 (32.0)	31 (31.6)	58 (31.2)
Bipolar Disorder	0 (0.0)	2 (2.7)	4 (4.1)	6 (3.2)
Anxiety	4 (30.8)	35 (46.7)	39 (39.8)	78 (41.9)
Schizophrenia	0 (0.0)	0 (0.0)	0 (0.0)	0 (0.0)
Personality Disorder	0 (0.0)	1 (1.3)	1 (1.0)	2 (1.1)
Other	2 (15.4)	8 (10.7)	7 (7.1)	17 (9.1)
No mental health diagnosis	6 (46.2)	33 (44.0)	51 (52.0)	90 (48.4)
History of psychotropic medications	5 (38.5)	26 (34.7)	35 (35.7)	66 (35.5)
Received MH counseling in the past year	7 (53.8)	35 (46.7)	30 (30.6)	72 (38.7)
Respondents would like to receive MH counseling	8 (61.5)	47 (62.7)	43 (43.9)	98 (52.7)
Respondents’ living areas on the 3rd of May when there was an order to evacuate Fort McMurray during the 2016 Wildfires?				
Fort McMurray	6 (66.7)	63 (86.3)	90 (94.7)	159 (89.8)
Other areas	3 (33.3)	10 (13.7)	5 (5.3)	18 (10.2)
Respondents who witnessed the burning of any homes or structures by the wildfires in Fort McMurray	5 (55.6)	60 (82.2)	83 (87.4)	148 (83.6)
Respondents who were fearful for their life or the lives of their friends or family on the day of evacuation	8 (88.9)	66 (90.4)	84 (88.4)	158 (89.3)
During the period of the evacuation order for Fort McMurray, how frequently did you watch television images about the devastation caused by the wildfires in Fort McMurray?				
Daily	6 (66.7)	56 (76.7)	80 (84.2)	142 (80.2)
Less than daily	3 (33.3)	10 (13.7)	10 (10.5)	23 (13.0)
Respondents did not watch TV images of the devastation	0 (0.0)	7 (9.6)	5 (5.3)	12 (6.8)
During the period of the evacuation order for Fort McMurray, how frequently did you read newspaper and internet articles related to the devastation caused by the wildfires in Fort McMurray?				
Daily	8 (88.9)	61 (83.6)	83 (87.4)	152 (85.9)
Less than daily	1 (11.1)	9 (12.3)	9 (9.5)	19 (10.7)
Respondents did not read newspaper or internet articles of the devastation	0 (0.0)	3 (4.1)	3 (3.2)	6 (3.4)
Property loss because of the wildfire in Fort McMurray				
Home was completely destroyed	1 (7.7)	12 (16.0)	15 (15.3)	28 (15.1)
Home suffered substantial smoke damage	1 (7.7)	10 (13.3)	11 (11.2)	22 (11.8)
Home suffered slight smoke damage	4 (30.8)	20 (26.7)	30 (30.6)	54 (29.0)
Car was completely destroyed	1 (7.7)	1 (1.3)	5 (5.1)	7 (3.8)
Business was completely destroyed	0 (0.0)	0 (0.0)	0 (0.0)	0 (0.0)
No loss	7 (53.8)	33 (44.0)	42 (42.9)	82 (44.1)
Do you live in the same house you lived in before the evacuation order came into effect?				
Yes	6 (66.7)	35 (47.9)	63 (67.0)	104 (59.1)
No; I live in a different house even though my previous home was not destroyed by the fire	3 (33.3)	25 (34.2)	18 (19.1)	46 (26.1)
No; I live in a different house because my previous home was destroyed by the flood	0 (0.0)	13 (17.8)	13 (13.8)	26 (14.8)
Did you receive sufficient support from family and friends after the evacuation order for Fort McMurray was declared?				
Yes, absolute support	5 (55.6)	49 (68.1)	62 (66.0)	116 (66.3)
Yes, some support	2 (22.2)	13 (18.1)	20 (21.3)	35 (20.0)
Yes, but only limited support	1 (11.1)	5 (6.9)	8 (8.5)	14 (8.0)
Not at all	1 (11.1)	5 (6.9)	4 (4.3)	10 (5.7)
Did you receive sufficient support from the Red Cross after the evacuation order for Fort McMurray was declared?				
Yes, absolute support	2 (22.2)	27 (37.5)	46 (48.9)	75 (42.9)
Yes, some support	3 (33.3)	24 (33.3)	28 (29.8)	55 (31.4)
Yes, but only limited support	3 (33.3)	7 (9.7)	12 (12.8)	22 (12.6)
Not at all	1 (11.1)	14 (19.4)	8 (8.5)	23 (13.1)
Did you receive sufficient support from the Government of Alberta after the evacuation order for Fort McMurray was declared?				
Yes, absolute support	1 (11.1)	20 (28.2)	36 (38.3)	57 (32.8)
Yes, some support	4 (44.4)	19 (26.8)	29 (30.9)	52 (29.9)
Yes, but only limited support	1 (11.1)	15 (21.1)	16 (17.0)	32 (18.4)
Not at all	3 (33.3)	17 (23.9)	13 (13.8)	33 (19.0)
Did you receive sufficient support from your insurers after the evacuation order for Fort McMurray was declared?				
Yes, absolute support	2 (22.2)	31 (43.7)	51 (54.3)	84 (48.3)
Yes, some support	3 (33.3)	14 (19.7)	25 (26.6)	42 (24.1)
Yes, but only limited support	3 (33.3)	6 (8.5)	10 (10.6)	19 (10.9)
Not at all	1 (11.1)	20 (28.2)	8 (8.5)	29 (16.7)
Respondents who received some counseling after returning to Fort McMurray after the wildfires	2 (22.0)	15 (21.1)	18 (19.1)	35 (20.1)

MH: mental health.

**Table 2:** Chi-square analyses of relationships between variables * and high-to-normal resilience and low resilience.

Variables	High-to-Normal Resilience	Low Resilience	Chi Square	*p*-Value
Gender				
Male	18 (75.0%)	6 (25.0%)		
Female	89 (60.5%)	58 (39.5%)	1.841	0.255
Age (Years)				
≤25	2 (22.2%)	7 (77.8%)		
26–40	41 (59.45)	28 (40.65)		
>40	64 (68.8%)	29 (31.2%)	8.098	0.015 *
Are you currently employed?				
No	2 (20.0%)	8 (80.0%)		
Yes	105 (65.2%)	56 (34.8%)	8.220	0.006 *
If employed, where?				
School boards	51 (65.4%)	27 (34.6%)		
Healthcare industry	6 (66.7%)	3 (33.3%)		
Keyano College	14 (70.0%)	6 (30.0%)		
Oil sands industry	7 (58.3%)	5 (41.7%)		
Municipal or government agency	10 (83.3%)	2 (16.7%)		
Other	16 (55.2%)	13 (44.8%)	3.474	0.641
Marital status				
Married/partnered/cohabiting	79 (63.7%)	45 (36.3%)		
Divorced/separated/widowed	11 (68.8%)	5 (31.2%)		
Single	17 (54.8%)	14 (45.2%)	1.121	0.634
Residence during the 2016 wildfire?				
No	7 (53.8%)	6 (46.2%)		
Yes	100 (63.3%)	58 (36.75)	0.458	0.557
Area of residence during the 2016 wildfire				
Timberlea	43 (61.4%)	27 (38.6%)		
Thickwood/Wood Buffalo/Persons Creek	32 (69.6%)	14 (30.4%)		
Other	25 (59.5%)	17 (40.5%)	1.140	0.575
Residence prior to 2016 wildfire				
Own home	79 (61.2%)	50 (38.8%)		
Renting	28 (66.7%)	14 (33.3%)	0.398	0.585
Current residence				
Own home	82 (60.3%)	54 (39.7%)		
Renting	25 (71.4%)	10 (28.6%)	1.474	0.247
Likely depression (PHQ-9 scale)				
Mild depression	73 (78.5%)	20 (21.5%)		
Moderate to high depression	32 (42.1%)	44 (57.9%)	23.538	<0.0010 *
Received mental health diagnosis (bipolar disorder)?				
Yes				
No	4 (66.7%)	2 (33.3%)	0.044	0.99
Likely anxiety disorder (GAD scale)				
Low anxiety	76 (79.2%)	20 (20.8%)		
Moderate-to-high anxiety	28 (39.4%)	43 (60.6%)	27.423	<0.0010 *
PTSD condition (PCL-C scale)				
Unlikely	78 (78.8%)	21 (21.2%)		
Likely	24 (78.9%)	41 (63.1%)	29.248	<0.0010 *
Received mental health diagnosis (alcohol abuse)?				
Yes	1 (33.3%)	2 (66.7%)		
No	106 (63.1%)	62 (36.9%)	1.115	0.557
Received mental health diagnosis (drug abuse)?				
Yes	1 (50.0%)	1 (50.0%)		
No	106 (62.7%)	63 (37.3%)	0.137	0.99
Received mental health diagnosis (personality disorder)?				
Yes	1 (100.0%)	0 (0.0%)		
No	106 (62.4%)	64 (37.6%)	0.602	0.99
Received mental health diagnosis (other diagnoses)?				
Yes	11 (64.7%)	6 (35.3%)		
No	96 (62.3%)	58 (37.7%)	0.037	0.99
Never received any mental health diagnosis?				
Yes	43 (48.3%)	46 (51.7%)		
No	64 (78.0%)	18 (22.0%)	16.112	<0.001 *
Are you on antidepressants?				
Yes	24 (44.4%)	30 (55.6%)		
No	83 (70.9%)	34 (29.1%)	11.076	0.001 *
Are you on antipsychotics?				
Yes	2 (50.0%)	2 (50.0%)		
No	105 (62.9%)	62 (37.1%)	0.276	0.631
I am not on any medications for mental health concerns				
Yes	29 (47.5%)	32 (52.5%)		
No	78 (70.9%)	32 (29.1%)	9.150	0.003 *
Have you received mental health counseling in the past?				
Yes	32 (49.2%)	33 (50.8%)		
No	75 (70.8%)	31 (29.2%)	7.971	0.006 *
Would you like to receive mental health counseling?				
Yes	40 (44.9%)	49 (55.1%)		
No	67 (81.7%)	15 (18.3%)	24.630	<0.001 *
Where did you live on the 3rd of May during evacuation for the 2016 wildfires?				
Fort McMurray	100 (64.5%)	55 (35.5%)		
Other	7 (43.8%)	9 (56.2%)	2.671	0.112
Did you witness the burning of homes during the wildfires?				
No	15 (55.6%)	12 (44.4%)		
Yes	92 (63.9%)	52 (36.1%)	0.674	0.516
Fearful for your life or those of family/friends during evacuation?				
No	17 (89.5%)	2 (10.5%)		
Yes	90 (59.2%)	62 (40.8%)	6.605	0.011 *
Frequency of watching TV on the wildfire devastation				
Daily	85 (62.0%)	52 (38.0%)		
<Daily	16 (69.6%)	7 (30.4%)		
I did not watch TV images of the devastation	6 (54.5%)	5 (45.5%)	0.799	0.689
Frequency of reading newspapers/articles on the wildfires				
Daily	89 (60.5%)	58 (39.5%)		
<Daily	14 (77.8%)	4 (22.2%)		
I did not read newspapers/articles on the wildfires	4 (66.7%)	2 (33.3%)	2.078	0.354
Lost property (home completely destroyed)				
No	92 (63.9%)	52 (36.1%)		
Yes	15 (55.6%)	12 (44.4%)	0.674	0.516
Lost property (home suffered substantial smoke damage)				
No	95 (63.8%)	54 (36.2%)		
Yes	12 (54.5%)	10 (45.5%)	0.695	0.481
Suffered no loss of property?				
No	49 (71.0%)	20 (29.0%)		
Yes	58 (56.9%)	44 (43.1%)	3.520	0.061
Did you live in the same house before the evacuation order?				
Yes	66 (66.0%)	34 (34.0%)		
No, I lived in a different house though my house was not destroyed	26 (57.8%)	19 (42.2%)		
No, I lived in a different house because my house was destroyed	14 (56.0%)	11 (44.0%)	1.398	0.497
Sufficient support from the Red Cross after evacuation?				
Yes, I had absolute support	53 (72.6%)	20 (27.4%)		
Yes, I had some support	28 (52.8%)	25 (47.2%)		
Yes, I had some limited support	12 (54.5%)	10 (45.5%)		
Not at all	12 (57.1%)	9 (42.9%)	6.111	0.107
Sufficient support from the Alberta Gov’t after evacuation?				
Yes, I had absolute support	41 (74.5%)	14 (25.5%)		
Yes, I had some support	29 (56.9%)	22 (43.1%)		
Yes, I had some limited support	19 (61.3%)	12 (38.7%)		
Not at all	16 (50.0%)	16 (50.0%)	6.215	0.101
Sufficient support from your insurers after evacuation?				
Yes, I had absolute support	59 (71.1%)	24 (28.9%)		
Yes, I had some support	19 (48.7%)	20 (51.3%)		
Yes, I had some limited support	11 (57.9%)	8 (42.1%)		
Not at all	16 (57.1%)	12 (42.9%)	6.251	0.100
Received counseling upon return to Fort McMurray?				
Yes	17 (51.5%)	16 (48.5%)		
No	88 (64.7%)	48 (35.3%)	1.964	0.168
Sufficient support from family/friends after evacuation?				
Yes, I had absolute support	77 (68.8%)	35 (31.2%)		
Yes, I had some support	17 (51.5%)	16 (48.5%)		
Yes, I had some limited support	4 (28.6%)	10 (71.4%)		
Not at all	7 (70.0%)	3 (30.0%)	10.631	0.012 *

NB: *p*-values ≤ 0.05 were denoted with *. PHQ-9: Patient Health Questionnaire. GAD-7: Generalized Anxiety Disorder 7-item Scale. PCL-C: Post-Traumatic Stress Disorder Checklist—civilian version. PTSD: post-traumatic stress disorder.

**Table 3:** Logistic regression model for respondents’ likelihood of presenting with high-to normal resilience and low resilience.

Variables	B	S.E.	Wald	df	*p* Value	Odds Ratio	95% CI for OR	
							Lower	Upper
Age								
≤25 years			6.236	2	0.044			
26 to 40 years	−3.131	1.572	3.966	1	0.046	0.044	0.002	0.952
>40 years	−3.704	1.593	5.404	1	0.020	0.025	0.001	0.559
Currently not employed	1.317	1.217	1.172	1	0.279	3.732	0.344	40.504
Are you on any medication (not on any medication)?	0.737	0.450	2.683	1	0.101	2.089	0.865	5.045
Received mental health counseling in the past year	−0.427	0.528	0.655	1	0.418	0.652	0.232	1.835
Would like to receive mental health counseling	0.817	0.517	2.492	1	0.114	2.263	0.821	6.236
Fearful for your life or those of friends/family during the evacuation	1.136	0.931	1.489	1	0.222	3.114	0.502	19.302
Lost property? (No loss of property in the fire)	−0.117	0.439	0.071	1	0.790	0.889	0.376	2.104
Sufficient support from your insurers after the evacuation order?								
Absolute support			1.687	3	0.640			
Some support	0.551	0.542	1.035	1	0.309	1.735	0.600	5.020
Limited support	0.220	0.721	0.093	1	0.760	1.246	0.303	5.120
No support	−0.246	0.661	0.139	1	0.710	0.782	0.214	2.855
Sufficient support from family and friends after the evacuation?								
Absolute support			2.204	3	0.531			
Some support	0.048	0.558	0.008	1	0.931	1.050	0.351	3.135
Limited support	0.877	0.850	1.065	1	0.302	2.403	0.455	12.702
No support	−0.808	0.973	0.690	1	0.406	0.446	0.066	3.003
Likely depression (PHQ-9 score)	0.233	0.493	0.223	1	0.637	1.262	0.480	3.316
Likely anxiety (GAD score)	0.646	0.504	1.646	1	0.200	1.909	0.711	5.125
Likely PTSD (PCL-C score)	1.046	0.504	4.312	1	0.038	2.845	1.060	7.632
Constant	0.182	1.640	0.012	1	0.912	1.200		

## Data Availability

The data of this study is available upon a reasonable request from the submitting author.
